# Integrated Serologic Surveillance of Population Immunity and Disease Transmission

**DOI:** 10.3201/eid2407.171928

**Published:** 2018-07

**Authors:** Benjamin F. Arnold, Heather M. Scobie, Jeffrey W. Priest, Patrick J. Lammie

**Affiliations:** University of California, Berkeley, California, USA (B.F. Arnold);; Centers for Disease Control and Prevention, Atlanta, Georgia, USA (H.M. Scobie, J.W. Priest, P.J. Lammie);; Task Force for Global Health, Atlanta (P.J. Lammie)

**Keywords:** antibodies, immunologic surveillance, communicable diseases, emerging, vaccination, malaria, neglected diseases, disease elimination, disease surveillance, neglected tropical diseases, transmission, immunity, multiplex antibody assays, United States, Cambodia

## Abstract

Antibodies are unique among biomarkers in their ability to identify persons with protective immunity to vaccine-preventable diseases and to measure past exposure to diverse pathogens. Most infectious disease surveillance maintains a single-disease focus, but broader testing of existing serologic surveys with multiplex antibody assays would create new opportunities for integrated surveillance. In this perspective, we highlight multiple areas for potential synergy where integrated surveillance could add more value to public health efforts than the current trend of independent disease monitoring through vertical programs. We describe innovations in laboratory and data science that should accelerate integration and identify remaining challenges with respect to specimen collection, testing, and analysis. Throughout, we illustrate how information generated through integrated surveillance platforms can create new opportunities to more quickly and precisely identify global health program gaps that range from undervaccination to emerging pathogens to multilayered health disparities that span diverse communicable diseases.

The potential to combine public health and environmental surveillance data with innovations in machine learning, statistical modeling, and data visualization has contributed to an emerging vision of precision public health, the idea that global health programs should use high-resolution data to guide interventions and direct scarce resources to those who would benefit most (*1*). Robust disease surveillance is a cornerstone of global health efforts that range from detecting emerging pathogens and epidemics to the control or elimination of vaccine-preventable diseases, HIV, malaria, and neglected tropical diseases (NTDs) (http://www.who.int/neglected_diseases/9789241564540/en/) (*2*–*4*). Most infectious disease surveillance maintains a single-disease focus. In this perspective, we encourage an integrated approach to surveillance of population immunity and infectious disease transmission. First, we argue that antibody-based methods provide a unique opportunity to augment and integrate surveillance across diverse global health initiatives. Second, we highlight multiple areas for synergy through integration, where the combined result will add more value to public health efforts than independent disease monitoring through vertical programs. Finally, we draw on innovations in laboratory and data science to suggest key ingredients for an integrated serologic surveillance (serosurveillance) platform. Throughout, we show how information generated through an integrated platform can create new opportunities to more quickly and precisely identify public health program gaps, and we draw on examples from many global health programs that use serosurveillance to target and monitor their efforts.

## Serology for Integrated Surveillance

Antibodies are unique among biomarkers in their ability to identify persons with protective immunity to vaccine-preventable diseases and to measure past exposure to diverse pathogens that range from viruses to bacteria, parasitic protozoa, and nematodes. All pathogens leave behind immunologic footprints in the form of antibodies that last for months to years and can be detected by testing dried blood spots or serum samples against panels of well-defined antigens. Antibody response provides an objective and sensitive way to uncover immunization coverage gaps or waning immunity to vaccine-preventable diseases (*5*–*7*) and monitor a population’s exposure to malaria (*8*), enteric pathogens (*9*–*12*), and many NTDs (*13*–*17*). Antibody response can also be a key tool to monitor epidemics, such as HIV (*18*) and emerging pathogens (*16*,*19*). Antibody levels reflect past exposure over a period of months to years, so cross-sectional surveys contain an immense amount of information about past vaccination and pathogen exposure (*8*,*20*).

Enteric pathogen incidence estimated from serosurveillance in European countries is 2–6 orders of magnitude higher than the rates estimated from standard case-based surveillance (*9*,*10*). This surprising statistic illustrates a key advantage of serosurveillance: most pathogen infections have asymptomatic presentation or mild symptoms, so reported clinical cases typically represent the tip of the iceberg in terms of actual infection. When measured in young children, antibody levels reveal recent often subclinical infections and, when measured on the population level, reflect the degree of disease endemicity, a characteristic that makes serosurveillance extremely useful for monitoring transmission interruption or recrudescence in malaria and NTD elimination settings (*8*,*21*). Measuring antibodies to vaccine-preventable diseases in young children can track improvements in infant vaccination through routine health services and complement program coverage data (*5*,*7*).

The development of multiplex serologic assays and the geographic overlap of many communicable diseases creates an opportunity to extend scarce global health resources beyond single-disease testing. For example, in the past 10 years Uganda completed >20 population-based serosurveys, including the Malaria Indicator Survey (2009, 2014); the AIDS Indicator Survey (2011); the Demographic and Health Surveys (2011, 2016); and NTD transmission assessment surveys in subsets of districts for lymphatic filariasis, onchocerciasis, schistosomiasis, soil-transmitted helminths, and trachoma (annually since 2008). Uganda’s highly monitored population illustrates how integrated serologic testing could potentially reduce the number of surveys required to monitor diverse global health programs. Alternatively, if disease-specific serosurveys remained in place but the number of antigens included in the tests were expanded, this strategy would increase the spatiotemporal resolution of information available across programs. In principle, integrated serosurveillance could include separate laboratory assays run on the same specimens, but multiplexed assays enable the highest efficiency in terms of cost and sample volume requirements. For example, multiplex bead assays on the Luminex platform (Luminex Corporation, Austin, TX, USA) enable the measurement of antibody responses to as many as 100 different antigens with just 1 μL of serum (*15*). When samples from a serosurvey in Cambodia were analyzed at the US Centers for Disease Control and Prevention (Atlanta, GA, USA), the cost of adding a tetanus toxoid–coupled bead to a multiplex assay with 19 other antigens was US $0.30/sample, and the total cost of the 20-plex assay was less than that of a double-antigen ELISA developed for tetanus (US $30/sample; P.J. Lammie, unpub. data) (*21*,*22*). In other surveys with the laboratory work performed in-country, our team at the Centers for Disease Control and Prevention estimated that the marginal cost of a 20-plex bead assay was US $20/sample (P.J. Lammie, unpub. data), similar to the cost of the 2 separate ELISAs for measles and rubella.

## Synergy through Integrated Serosurveillance

### Uncovering Public Health Program Gaps and Overlap in Disease Exposure

**Figure 1:**
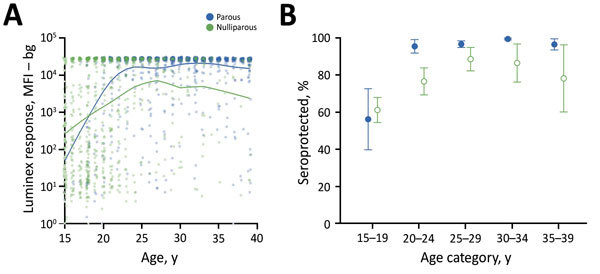
Tetanus toxoid antibody response of 2,150 women, by age and reproductive status, Cambodia, 2012. Specimens were collected from women who had (parous) and had not (nulliparous) previously given birth in a nationally representative immunization coverage survey (*23*) and measured by using the Luminex platform (Luminex Corporation, Austin, TX, USA). A) Mean antibody response. B) Percentage and 95% CIs of women seroprotected (>100 MFI) (*23*). We estimated age-dependent means and seroprevalence using previously described methods (*24*). Data set and computational notebook are available through the Open Science Framework (https://osf.io/2kr8b). MFI – bg, mean fluorescence intensity minus background.

Age-structured serosurveys can provide information about age-specific immunity gaps for vaccine-preventable diseases that result from missed vaccination or waning immunity (*7*,*20*). For example, immunizing reproductive-age women through antenatal care visits is needed for boosting tetanus seroprotection and preventing neonatal tetanus in lower-income countries. Serologic data from Cambodia revealed the success of that country’s maternal and neonatal tetanus elimination program and identified that additional effort is still needed to reach young, first-time mothers (Figure 1, panels A, B) (*25*). This example illustrates how analyses of single antibodies can reveal public health program gaps, but the most novel and synergistic opportunities will most likely emerge from concurrent measurement of antibody responses to a broad pathogen panel.

**Figure 2:**
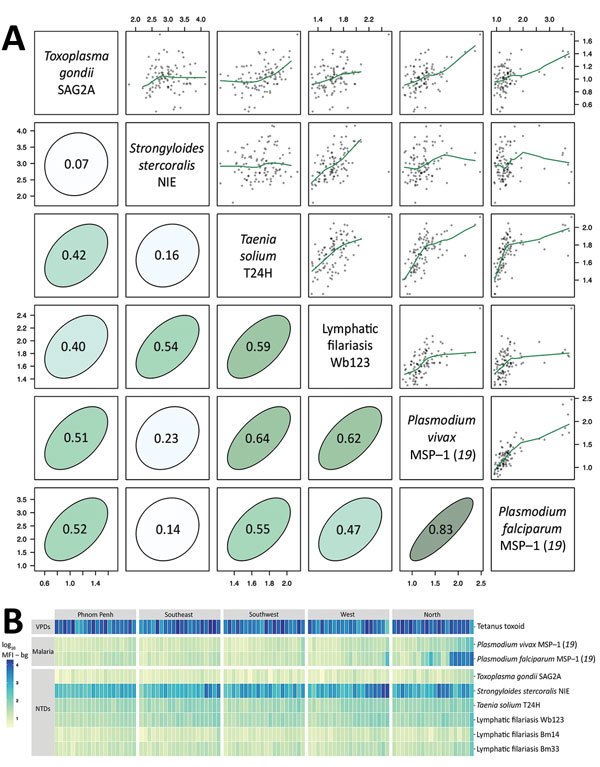
Antibody response to tetanus toxoid and causative agents of malaria and NTDs measured by multiplex bead assay among 2,150 women, Cambodia, 2012. Specimens were measured by using the Luminex platform (Luminex Corporation, Austin, TX, USA) (*25*). A) Relationship between pairs of antibodies measured by mean antibody response (log_10_ MFI – bg) in each of the 100 sampling clusters. Scatter plots include nonparametric locally weighted regression fits trimmed to reduce edge effects. Correlation ellipses depict the strength of the association on the basis of the Pearson correlation (r estimates). Both axes indicate mean antibody response. B) Heatmap of mean antibody response to tetanus toxoid and pathogens that cause malaria and NTDs in 100 sampling clusters stratified by region and then sorted by mean antibody response. Data set and computational notebook are available through the Open Science Framework (https://osf.io/2kr8b). MFI – bg, mean fluorescence intensity minus background; MSP, merozoite surface protein; NTDs, neglected tropical diseases; SAG2A, surface antigen 2A; VPDs, vaccine-preventable diseases.

The simultaneous measurement of antibody responses to multiple pathogens could uncover populations with high exposure to multiple infectious diseases and potentially multilayered health disparities. This, in turn, should create opportunities to integrate program delivery rather than relying on multiple vertical programs separately delivering interventions, a paradigm shift that aligns with calls for integrated global health systems (*26*). High pairwise correlation (r>0.5) of mean antibody responses to different antigens across geographic clusters in the Cambodia serosurvey suggests overlap in disease exposure and transmission (Figure 2, panel A). Cluster-level mean antibody responses reveal regional differences in exposure but also provide multilayered information that could help identify opportunities for coordinated response. For example, clusters in the western and northern regions of Cambodia with the highest *Plasmodium falciparum* antibody levels also have high antibody levels to the causative agent of lymphatic filariasis (*Wuchereria bancrofti*) and *Strongyloides stercoralis* but low antibody levels to tetanus toxoid (Figure 2, panel B, far right columns under West and North).

The Cambodia survey illustrates how a relatively small panel of antigens in a multiplex assay can extend the value of blood collection, and including a broader panel (Technical Appendix Table) could provide even more information in future surveys. Similar measurements among young children would more accurately reflect recent exposure than measurement in adults, which would reflect both short-term and long-lived antibody responses (*27*). For pathogens with exposure that begins at birth, measurements in young children capture the key period of age-dependent antibody acquisition that differentiates transmission across populations (*24*). High-resolution maps of overlapping serologic responses estimated through geostatistical predictive algorithms could contribute information to integrated surveillance-and-response systems (discussed later) (*27*,*28*). If extended in this way, multiplex serologic testing could create new opportunities for coordinated and appropriate response across diverse diseases.

### New Opportunities to Measure Health Burden and Progress toward United Nations Sustainable Development Goals

Integrated serosurveillance would dramatically expand our knowledge of pathogens that are not currently part of routine surveillance in low- and middle-income countries. Serologic data combined with mathematical models were critical to developing global burden of disease estimates for rubella and hepatitis (*7*). Similar efforts could augment burden of disease estimates for enteric pathogens, NTDs, and other pathogens that have well-defined antigen targets but lack population-based information about incidence. For example, including antibody responses to diverse enteric pathogens in population-based surveys could improve burden of disease estimates and provide an objective indicator through which we could view progress toward achieving related United Nations sustainable development goals 3.3 (improving health and well-being target 3, which includes reducing waterborne diseases) and 6 (universal access to clean water and sanitation) (*11*,*29*).

### New Opportunities for Immunoepidemiology

Integrating multiplex assays into geolocated, population-based surveys would enable an unprecedented characterization of population-level, multipathogen immunologic profiles. Large-scale studies drawing on this resource could lead to new biological insights about how disease occurrence is interrelated and provide clues for future experimental studies to examine mechanisms of immune modulation (*19*,*30*). For example, helminths infect >800 million persons worldwide, and growing evidence suggests these pathogens influence malaria infection, HIV infection, vaccine effectiveness, and even fecundity through complex immunoregulatory pathways (*31*–*34*). As another example, studies have linked seasonal El Niño atmospheric conditions to profound shifts in environmentally mediated infections, such as regional-scale cholera epidemics and mosquitoborne pathogen transmission (e.g., malaria, arboviruses) (*35*,*36*). Antibody titers in young children are a sensitive measure of recent pathogen exposure and have even been shown to fluctuate with seasonal changes in malaria transmission (*24*). Because antibody levels reflect symptomatic and asymptomatic infections, measuring antibody responses could reveal a more complete picture of seasonally driven changes in transmission compared with case-based surveillance.

## Path toward Integrated Serosurveillance

### Specimen Collection

Serum samples and dried blood spots are already collected for programs such as the Demographic and Health Surveys, Malaria and AIDS Indicator Survey, and NTD transmission assessment surveys. With small changes in protocol, samples from these platforms could be used for routine multiplex testing. In most cases, protocol changes will amount to ensuring adequate consent to enable broad-based serologic testing and sending specimens to regional or national laboratories with the capacity for multiplex testing. Broader multiplex testing of routine clinical samples could supplement the information available through these population-based surveys, and expanded testing within other focused serologic surveys could contribute as well (*16*,*25*,*37*). In all cases, adequate safeguards for participant privacy and biosafety will be essential and should follow similar protocols already in place for existing periodic serosurveys (e.g., Malaria and AIDS Indicator Surveys) and global laboratory testing networks (*38*). Dried blood spots collected on filter paper from finger or heel pricks are easy to collect, transport, and preserve, and antibody results from dried blood spot eluates are comparable with those obtained directly from serum samples for malaria, some helminths, and a broad range of viral pathogens, including vaccine-preventable diseases and dengue (*39*–*42*). Additional validation studies would help ensure that field protocols are adequate for the broad set of antigens envisioned in an integrated platform (Technical Appendix Table).

A challenge to integrated serosurveillance is that different diseases have different ideal populations for monitoring, governed by disease-specific transmission dynamics. For example, children are a focus of most NTD and vaccine-preventable disease monitoring, but teens and adults are most relevant in surveillance of HIV, tetanus protection, and in some cases malaria (e.g., forest workers). Enteric pathogen transmission is often so intense in low-income settings that most population-level heterogeneity in antibody response occurs in the first few years of life (*24*), an age range that is only partly represented in population-based samples. Moreover, HIV surveillance programs have demonstrated that sampling methods that extend beyond population-based surveys and passive clinical surveillance are needed to capture the highest risk groups, and similar methods will probably be necessary for malaria and other pathogens in elimination settings (*43*). Aligning optimal sampling methods across diseases is a substantive challenge, but adaptive geostatistical design methods (*44*) could potentially start with the information collected during the initial integrated, population-based serosurvey and then augment the information needed for disease-specific control priorities through follow-on, enrichment sampling of persons in ideal age ranges and with high-risk characteristics.

### Specimen Testing

From a large set of potential antigens (Technical Appendix Table), each survey could include a subset tailored to specific disease monitoring objectives, while new antigens could be added in response to emerging threats. For example, including a recombinant chikungunya virus antigen in multiplex testing of an existing filariasis surveillance cohort tracked the virus’s introduction to Haiti during 2013–2014 (*16*). Technologic advances on bead-based platforms, such as Luminex, will further extend the number of antigens included in a single assay, and new antigen discovery through high-throughput screening studies will further enrich integrated platforms (*45*). A current limitation of the technology is that individual antigen-coupled beads are generally not yet commercially available; future commercial development of these reagents would enable better standardization across laboratories. Bead-antigen coupling and bead degradation can introduce variability into multiplex assays, so the use of standardized operating procedures and a consistent bead preparation for each survey are essential. Another limitation is that global reference serum standards to translate arbitrary units in serologic assays into international unit values are mainly limited to vaccine-preventable diseases. Reference standards for other pathogens, such as non–*P. falciparum* malarias and NTDs, would add immense value because they would enable direct comparison of quantitative results across surveys and laboratories. Finally, antibody measurements will be most useful if they are integrated into a coordinated repository and testing platform, such as the recently proposed World Serology Bank (*19*), which could further streamline laboratory protocols, accessibility to reagents, and funding. Surveillance laboratory networks for vaccine-preventable diseases provide a model for how globally standardized testing can work in practice (*38*).

### Analysis Pipelines to Provide Actionable Information

Integrated serosurveillance will only reduce infectious disease transmission if it translates into actionable information and triggers a response by effective programs. In this context, information must be timely, accurate, and high resolution to be actionable from a programmatic perspective. Generating actionable information at spatial scales much smaller than national or district levels is commonplace in high-income countries and should be a near-term, attainable goal for the rest of the world (*1*). Efforts in precision global health exemplify how information could be integrated across serosurveys in space and time; high-resolution estimates of child growth failure, measles immunization gaps, and malaria mortality rates show how advances in computation, modeling, and data science have accelerated the development of new pipelines for processing, analysis, and visualization to support precision public health that spans from village to continental scales (*46*–*48*). Integrated serosurveillance will be best positioned to contribute to this endeavor if serology measurements flow into efficient data pipelines and analysis methods are general enough to accommodate diverse pathogens. The breadth of antigens incorporated into multiplex assays (Technical Appendix Table) means that a single integrated serosurveillance platform could potentially generate spatially explicit estimates of vaccine immunity, malaria transmission, NTD transmission, and HIV incidence.

For antigenically stable pathogens, force of infection can be estimated from cross-sectional surveys with general methods that range in approach from mathematical modeling to nonparametric survival analyses (*20*). For infections that lead to partial or transient immunity, it might be possible to extend existing approaches to estimate force of infection among young children, provided that antibody levels remain sufficiently elevated for multiple years. The distribution of infectious disease transmission in populations is often highly heterogeneous in space, and for this reason, malaria and NTD elimination efforts have led to the development of sophisticated data pipelines that aggregate, analyze, and map surveillance data with rapid updates (*49*,*50*). Mapping antibody response is a relatively underexploited opportunity, and existing platforms could be extended to include multiplex serologic data. Combining antibody levels, seroprevalence, or force of infection estimates with geospatial prediction algorithms could lead to high-resolution, richly layered maps of infectious disease exposure and immunity that would be an immense resource for precision guidance of global public health programs.

### Financing

Integrated serosurveillance will generate information that is a global public good (*26*), and international financing will be essential to support coordination across programs for specimen storage, testing, analysis, and reporting. Coordinated financing would also help ensure harmonization across each step in the collection, testing, and analysis pipeline. As the global community prepares for a world after polio eradication, extending the polio surveillance infrastructure and integrating surveillance across vaccine-preventable diseases has been proposed (*38*). World Health Organization reference laboratory networks for vaccine-preventable diseases already support serologic testing for measles, rubella, yellow fever, and Japanese encephalitis and have the technical capacity to support high-throughput serologic assays. Additional financial support that builds from this existing laboratory infrastructure could reinforce investments that are already in place and extend the serologic testing platform beyond vaccine-preventable diseases. In an analogous example, the World Health Organization’s Global Rotavirus Laboratory Network tests fecal specimens for the presence of >20 enteric pathogens other than rotavirus using multiplex molecular assays (*38*). For data analysis and synthesis, the Institute for Health Metrics and Evaluation’s Local Burden of Disease Project provides an example of how coordinated financing can be used to aggregate, analyze, and disseminate information through sophisticated data pipelines to support diverse precision public health efforts (http://www.healthdata.org/lbd).

## Conclusions

Integrated serologic surveillance is a concept with enormous potential. If deployed at scale through existing surveys and integrated into existing data pipelines, multiplex antibody testing will enable the global community to more quickly identify and respond to public health gaps, including undervaccination, emerging infectious diseases, persistent areas of high transmission, and recrudescence of NTDs or malaria in elimination settings. The key elements for building an integrated serosurveillance platform are within reach, and in our view, it is time to move beyond proof-of-concept studies toward a systematic, integrated platform.

Technical Appendix Examples of antigens included in multiplex bead assays.
