# Supplementary material for: Integrated Serologic Surveillance of Population Immunity and Disease Transmission
Source: Emerg Infect Dis. 2018 Jul;24(7):1188–94. doi: 10.3201/eid2407.171928 (PMC6038749; doi:10.3201/eid2407.171928)
Supplement: Technical Appendix — Examples of antigens included in multiplex bead assays. [file 17-1928-Techapp-s1.pdf]

Article DOI: https://doi.org/10.3201/eid2407.171928

Integrated Serologic Surveillance of Population Immunity and Disease Transmission

Technical Appendix

Technical Appendix Table. Examples of antigens that have been included in multiplex bead assays*

Pathogen or disease	Antigen
Vaccine preventable diseases	
Measles	Whole virus (*1*)
Rubella	Inactivated whole virus (*1*)
Mumps	Inactivated whole virus (*1*)
Varicella-zoster	Whole virus (*1*)
Diphtheria	Diphtheria toxoid (*2*)
Tetanus	Tetanus toxoid (*3–5*)
Pertussis	Pertussis toxin, FHA, Prn (*3*,*4*)
Human papillomavirus	E6, E7, L1 proteins (*6*)
HIV	
HIV-1	p66, gp120, gp160, gp41 (*7*)
Malaria	
Plasmodium falciparum	MSP-1 (*7*), AMA-1, CSP (*8–10*)
Plasmodium vivax	MSP-1 (*7*,*8*)
Malaria (multiple species)	Multiple proteins and peptides (*11*,*12*)
Neglected tropical diseases	
Lymphatic filariasis (*Wuchereria bancrofti*)	Bm33, Bm14, Wb123 (*13*)
River blindness (*Onchocerca volvulus*)	Ov16, Ov17, Ov33 (*14*)
Trachoma (*Chlamydia trachomatis*)	Pgp3, CT694 (*15*)
Yaws (*Treponema pallidum*)	rp17, TmpA (*16*)
Schistosomiasis (*Schistosoma mansoni*)	SEA, Sm25, SERPIN (*17*,*18*)
Strongyloidiasis (*Strongyloides stercoralis*)	NIE (*8*,*19*)
Cysticercosis (*Taenia solium*)	T24H (*8*)
Ascariasis (*Ascaris* spp.)	AsHb†
Dengue virus	Dengue 2 VLP, dengue 3 VLP (*20*)
Chikungunya virus	Envelope E1 protein (*20*)
Multiple viruses (including Ebola and Lassa)	Inactivated whole virus (*21*)
Leishmania donovani	KRP42 (*22*)
Toxocariasis (*Toxocara canis*)	Tc-CTL-1, Tc-TES-26 (*23*)
Enteric pathogens	
Cryptosporidium parvum	Cp17, Cp23, CpP2 (*24*)
Giardia intestinalis	VSP1–5, VSP7 (*24*)
Entamoeba histolytica	LecA, C-IgL (*22*,*25*)
Campylobacter jejuni	p18, p39†
Salmonella enterica serotype typhimurium	LPS group B (*26*)
Salmonella enterica serotype enteriditis	LPS group D†
Enterotoxigenic *Escherichia coli*	Heat labile toxin β subunit (*26*)
Vibrio cholerae	Cholera toxin β subunit (*22*)
Norovirus	VLP GI.4, VLP GII.4 New Orleans (*26*)
Other	
Heliobacter pylori	Multiple (*27*)
Mycobacterium tuberculosis	Multiple (*28*)
Toxoplasma gondii	SAG1, SAG2A (*22*,*29*)
Babesia microti	Bm17N, Bm9c (*30*)

*AMA-1, apical membrane antigen 1; AsHb, *Ascaris suum* hemoglobin; C-Igl, C-terminal of *Entamoeba histolytica* intermediate subunit of galactose; CSP, circumsporozoite protein; FHA, filamentous hemagglutinin; KRP42, kinesin-related protein 42; LPS, lipopolysaccharide; MSP-1, merozoite surface protein 1; prn, pertactin; SAG, surface antigen; SEA, soluble egg antigen; SERPIN, serine protease inhibitor; Tc-CTL-1, *Toxocara canis* control antigen 1; Tc-TES-26, *Toxocara canis* excretory/secretory antigen of 26 kDa; TmpA, treponemal membrane protein A; VLP, virus-like particle; VSP, variant-specific surface protein.
†Details available upon request (P.J. Lammie, unpub. data).

References

1. Smits GP, van Gageldonk PG, Schouls LM, van der Klis FR, Berbers GA. Development of a bead-based multiplex immunoassay for simultaneous quantitative detection of IgG serum antibodies against measles, mumps, rubella, and varicella-zoster virus. Clin Vaccine Immunol. 2012;19:396–400. PubMed http://dx.doi.org/10.1128/CVI.05537-11

2. van Gageldonk PGM, von Hunolstein C, van der Klis FRM, Berbers GAM. Improved specificity of a multiplex immunoassay for quantitation of anti-diphtheria toxin antibodies with the use of diphtheria toxoid. Clin Vaccine Immunol. 2011;18:1183–6. PubMed http://dx.doi.org/10.1128/CVI.05081-11

3. van Gageldonk PGM, van Schaijk FG, van der Klis FR, Berbers GAM. Development and validation of a multiplex immunoassay for the simultaneous determination of serum antibodies to *Bordetella pertussis*, diphtheria and tetanus. J Immunol Methods. 2008;335:79–89. PubMed http://dx.doi.org/10.1016/j.jim.2008.02.018

4. Reder S, Riffelmann M, Becker C, Wirsing von König CH. Measuring immunoglobulin G antibodies to tetanus toxin, diphtheria toxin, and pertussis toxin with single-antigen enzyme-linked immunosorbent assays and a bead-based multiplex assay. Clin Vaccine Immunol. 2008;15:744–9. PubMed http://dx.doi.org/10.1128/CVI.00225-07

5. Scobie HM, Mao B, Buth S, Wannemuehler KA, Sørensen C, Kannarath C, et al. Tetanus immunity among women aged 15 to 39 years in Cambodia: a national population-based serosurvey, 2012. Clin Vaccine Immunol. 2016;23:546–54. PubMed http://dx.doi.org/10.1128/CVI.00052-16

6. Waterboer T, Sehr P, Michael KM, Franceschi S, Nieland JD, Joos TO, et al. Multiplex human papillomavirus serology based on in situ-purified glutathione S-transferase fusion proteins. Clin Chem. 2005;51:1845–53. PubMed http://dx.doi.org/10.1373/clinchem.2005.052381

7. Curtis KA, Kennedy MS, Charurat M, Nasidi A, Delaney K, Spira TJ, et al. Development and characterization of a bead-based, multiplex assay for estimation of recent HIV type 1 infection. AIDS Res Hum Retroviruses. 2012;28:188–97. PubMed http://dx.doi.org/10.1089/aid.2011.0037

8. Priest JW, Jenks MH, Moss DM, Mao B, Buth S, Wannemuehler K, et al. Integration of multiplex bead assays for parasitic diseases into a national, population-based serosurvey of women 15-39 years of age in Cambodia. PLoS Negl Trop Dis. 2016;10:e0004699. PubMed http://dx.doi.org/10.1371/journal.pntd.0004699

9. Arnold BF, Priest JW, Hamlin KL, Moss DM, Colford JM Jr, Lammie PJ. Serological measures of malaria transmission in Haiti: comparison of longitudinal and cross-sectional methods. PLoS One. 2014;9:e93684. PubMed http://dx.doi.org/10.1371/journal.pone.0093684

10. Rogier E, Wiegand R, Moss D, Priest J, Angov E, Dutta S, et al. Multiple comparisons analysis of serological data from an area of low *Plasmodium falciparum* transmission. Malar J. 2015;14:436. PubMed http://dx.doi.org/10.1186/s12936-015-0955-1

11. Kerkhof K, Canier L, Kim S, Heng S, Sochantha T, Sovannaroth S, et al. Implementation and application of a multiplex assay to detect malaria-specific antibodies: a promising tool for assessing malaria transmission in Southeast Asian pre-elimination areas. Malar J. 2015;14:338. PubMed http://dx.doi.org/10.1186/s12936-015-0868-z

12. Koffi D, Touré AO, Varela M-L, Vigan-Womas I, Béourou S, Brou S, et al. Analysis of antibody profiles in symptomatic malaria in three sentinel sites of Ivory Coast by using multiplex, fluorescent, magnetic, bead-based serological assay (MAGPIX™). Malar J. 2015;14:509. PubMed http://dx.doi.org/10.1186/s12936-015-1043-2

13. Hamlin KL, Moss DM, Priest JW, Roberts J, Kubofcik J, Gass K, et al. Longitudinal monitoring of the development of antifilarial antibodies and acquisition of *Wuchereria bancrofti* in a highly endemic area of Haiti. PLoS Negl Trop Dis. 2012;6:e1941. PubMed http://dx.doi.org/10.1371/journal.pntd.0001941

14. Feeser KR, Cama V, Priest JW, Thiele EA, Wiegand RE, Lakwo T, et al. Characterizing reactivity to *Onchocerca volvulus* antigens in multiplex bead assays. Am J Trop Med Hyg. 2017;97:666–72. PubMed http://dx.doi.org/10.4269/ajtmh.16-0519

15. Goodhew EB, Priest JW, Moss DM, Zhong G, Munoz B, Mkocha H, et al. CT694 and pgp3 as serological tools for monitoring trachoma programs. PLoS Negl Trop Dis. 2012;6:e1873. PubMed http://dx.doi.org/10.1371/journal.pntd.0001873

16. Cooley GM, Mitja O, Goodhew B, Pillay A, Lammie PJ, Castro A, et al. Evaluation of multiplex-based antibody testing for use in large-scale surveillance for yaws: a comparative study. J Clin Microbiol. 2016;54:1321–5. PubMed http://dx.doi.org/10.1128/JCM.02572-15

17. Won KY, Kanyi HM, Mwende FM, Wiegand RE, Goodhew EB, Priest JW, et al. Multiplex serologic assessment of schistosomiasis in western Kenya: antibody responses in preschool aged children as a measure of reduced transmission. Am J Trop Med Hyg. 2017;96:1460–7. PubMed http://dx.doi.org/10.4269/ajtmh.16-0665

18. Tanigawa C, Fujii Y, Miura M, Nzou SM, Mwangi AW, Nagi S, et al. Species-specific serological detection for schistosomiasis by serine protease inhibitor (SERPIN) in multiplex assay. PLoS Negl Trop Dis. 2015;9:e0004021. PubMed http://dx.doi.org/10.1371/journal.pntd.0004021

19. Rascoe LN, Price C, Shin SH, McAuliffe I, Priest JW, Handali S. Development of Ss-NIE-1 recombinant antigen based assays for immunodiagnosis of strongyloidiasis. PLoS Negl Trop Dis. 2015;9:e0003694. PubMed http://dx.doi.org/10.1371/journal.pntd.0003694

20. Poirier MJP, Moss DM, Feeser KR, Streit TG, Chang G-JJ, Whitney M, et al. Measuring Haitian children's exposure to chikungunya, dengue and malaria. Bull World Health Organ. 2016;94:817–825A. PubMed http://dx.doi.org/10.2471/BLT.16.173252

21. O'Hearn AE, Voorhees MA, Fetterer DP, Wauquier N, Coomber MR, Bangura J, et al. Serosurveillance of viral pathogens circulating in West Africa. Virol J. 2016;13:163. PubMed http://dx.doi.org/10.1186/s12985-016-0621-4

22. Fujii Y, Kaneko S, Nzou SM, Mwau M, Njenga SM, Tanigawa C, et al. Serological surveillance development for tropical infectious diseases using simultaneous microsphere-based multiplex assays and finite mixture models. PLoS Negl Trop Dis. 2014;8:e3040. PubMed http://dx.doi.org/10.1371/journal.pntd.0003040

23. Anderson JP, Rascoe LN, Levert K, Chastain HM, Reed MS, Rivera HN, et al. Development of a Luminex bead based assay for diagnosis of toxocariasis using recombinant antigens Tc-CTL-1 and Tc-TES-26. PLoS Negl Trop Dis. 2015;9:e0004168. PubMed http://dx.doi.org/10.1371/journal.pntd.0004168

24. Priest JW, Moss DM, Visvesvara GS, Jones CC, Li A, Isaac-Renton JL. Multiplex assay detection of immunoglobulin G antibodies that recognize *Giardia intestinalis* and *Cryptosporidium parvum* antigens. Clin Vaccine Immunol. 2010;17:1695–707. PubMed http://dx.doi.org/10.1128/CVI.00160-10

25. Moss DM, Priest JW, Hamlin K, Derado G, Herbein J, Petri WA Jr, et al. Longitudinal evaluation of enteric protozoa in Haitian children by stool exam and multiplex serologic assay. Am J Trop Med Hyg. 2014;90:653–60. PubMed http://dx.doi.org/10.4269/ajtmh.13-0545

26. Arnold BF, van der Laan MJ, Hubbard AE, Steel C, Kubofcik J, Hamlin KL, et al. Measuring changes in transmission of neglected tropical diseases, malaria, and enteric pathogens from quantitative antibody levels. PLoS Negl Trop Dis. 2017;11:e0005616. PubMed http://dx.doi.org/10.1371/journal.pntd.0005616

27. Gao L, Michel A, Weck MN, Arndt V, Pawlita M, Brenner H. *Helicobacter pylori* infection and gastric cancer risk: evaluation of 15 *H. pylori* proteins determined by novel multiplex serology. Cancer Res. 2009;69:6164–70. PubMed http://dx.doi.org/10.1158/0008-5472.CAN-09-0596

28. Khan IH, Ravindran R, Yee J, Ziman M, Lewinsohn DM, Gennaro ML, et al. Profiling antibodies to *Mycobacterium tuberculosis* by multiplex microbead suspension arrays for serodiagnosis of tuberculosis. Clin Vaccine Immunol. 2008;15:433–8. PubMed http://dx.doi.org/10.1128/CVI.00354-07

29. Priest JW, Moss DM, Arnold BF, Hamlin K, Jones CC, Lammie PJ. Seroepidemiology of *Toxoplasma* in a coastal region of Haiti: multiplex bead assay detection of immunoglobulin G antibodies that recognize the SAG2A antigen. Epidemiol Infect. 2015;143:618–30. PubMed http://dx.doi.org/10.1017/S0950268814001216

30. Priest JW, Moss DM, Won K, Todd CW, Henderson L, Jones CC, et al. Multiplex assay detection of immunoglobulin G antibodies that recognize *Babesia microti* antigens. Clin Vaccine Immunol. 2012;19:1539–48. PubMed http://dx.doi.org/10.1128/CVI.00313-12
